# iTRAQ-Based Proteomics Investigation of Aqueous Humor from Patients with Coats' Disease

**DOI:** 10.1371/journal.pone.0158611

**Published:** 2016-07-14

**Authors:** Qiong Yang, Hai Lu, Xudong Song, Songfeng Li, Wenbin Wei

**Affiliations:** Beijing Tongren Eye Center, Beijing Tongren Hospital, Capital Medical University, Beijing Key Laboratory of Intraocular Tumor Diagnosis and Treatment, Beijing Ophthalmology and Visual Science Key Lab, Beijing, 100730, China; Shiraz University, ISLAMIC REPUBLIC OF IRAN

## Abstract

**Background:**

Coats' disease is an uncommon form of retinal telangiectasis, and the identification of novel proteins that contribute to the development of Coats' disease is useful for improving treatment efficacy. Proteomic techniques have been used to study many eye diseases; however, few studies have used proteomics to study the development of Coats' disease.

**Methods:**

Isobaric tagging for relative and absolute protein quantification (iTRAQ) was employed to screen differentially expressed proteins (DEPs) in the aqueous humor (AH) between stage 3A patients (n = 8), stage 3B patients (n = 14), stage 4 patients (n = 2) and control patients (n = 20). Differentially co-expressed proteins (DCPs) were present in all three stages of Coats' disease and were considered disease-specific proteins. These proteins were further analyzed using Gene Ontology (GO) functional annotations.

**Results:**

A total of 819 proteins were identified in the AH, 222 of which were significantly differentially expressed (fold change > 2 and P < 0.05) in the samples from at least one stage of Coats' disease. Of the DEPs, 46 were found among all three stages of Coats' disease and the controls; therefore, they were considered Coats' disease-specific proteins (DCPs). A GO classification analysis indicated that the DCPs were closely related to structural molecule activity, cell adhesion molecule binding and receptor binding. Western blotting confirmed the expression levels of haptoglobin and apolipoprotein C-I were significantly up-regulated in Coats’ disease.

**Conclusions:**

The 46 Coats' disease-specific proteins may provide additional insights into the mechanism of Coats' disease and represent potential biomarkers for identifying individuals with Coats' disease.

## Introduction

Coats' disease is a form of abnormal telangiectasia that is primarily characterized by aneurysms of retinal vessels and excessive production of yellowish intraretinal and subretinal exudates. Coats’ disease predominantly affects boys or young men [1], can cause retinal detachment and severe visual loss, and has been classified into five stages: stage 1, stage 2, stage 3, stage 4 and stage 5 [2]. Presently, the main therapeutic strategy for Coats' disease is direct coagulation of the abnormal vessels using techniques such as laser photocoagulation [3, 4]. However, direct coagulation eventually increases the subretinal exudates, which promotes secondary retinal detachment [1] as well as complications related to exudative retinal detachment [5]. Thus, this technique is not effective for cases with severe exudative changes [6].

In recent decades, significant efforts have been devoted to investigating the pathogenesis of Coats' disease; however, the cause remains largely unknown. Recently, proteomics have been widely used to obtain abundant information on individual proteins in various ocular diseases, including cataracts and retinopathy [7–10]. In combination with mass spectrometry, isobaric tagging for relative and absolute protein quantification (iTRAQ) has recently been demonstrated to be a sensitive quantitative proteomic method for high-throughput protein identification and quantification [11]. The AH is an intraocular fluid that plays an important role in supplying nutrients and removing metabolic waste from the avascular tissues of the eye [12]. Accumulating evidence suggests that certain proteins in the AH are closely correlated with the mechanisms underlying many eye disorders [13–15]. Studies have shown that vascular endothelial growth factor (VEGF), an important intraocular cytokine, is significantly up-regulated in AH samples from patients with increasingly severe Coats' disease [16–18]. In addition, the levels of nitric oxide in the AH of patients with Coats' disease are elevated, indicating proteins involve in nitric oxide metabolism may affect Coats’ disease development [19]. These proteins, which include antioxidant and immunoregulatory proteins, are essential for regulating homeostasis and may play a crucial role in the pathogenesis of Coats' disease [20].

In this study, we used iTRAQ to conduct comparative proteome profiling of the AH samples between patients with three different stages of Coats’ disease and control patients to identify disease-specific proteins in the AH. Our findings identified potential AH biomarkers that could be used to predict the development of Coats' disease. In addition, our findings may contribute to a better understanding of the molecular events involved in the pathogenesis of Coats' disease.

## Materials and Methods

### Ethics statement

All patients provided written informed consent prior to participation, and the clinical study was approved by the Medical Ethics Committee of Capital Medical University.

### Patient eligibility and recruitment

A total of 44 participants that included 20 controls (CK, senile cataract patients) and 24 patients with Coats' disease were recruited through the medical ethics committee of Capital Medical University in Beijing, China from 2014 to 2015. The participants provided written consent for the donation and use of their aqueous humor samples. All subjects met the inclusion criteria, which included the absence of ocular disease other than Coats' disease and a lack of systemic antimetabolite or immunosuppressant usage. Samples from patients with Coats' disease were classified as stage 3A (n = 8), 3B (n = 14) and 4 (n = 2) according to previously reported classification standards [1]. Unfortunately, no stage 5 patient was found during the sample collection procedure of our investigation. Relative information on the participants, including their gender and age, are listed in Table 1.

**Table 1:** Summary of the human AH samples from the patients with Coats' disease and the controls without Coats' disease.

Group/Stage	Specimen	Average age (year)	Gender (F: female, M: male)
control	20	68.15 ± 10.27	9 F, 11 M
3A	8	7.38 ± 3.11	1 F, 7 M
3B	14	6.93 ± 3.17	1 F, 13 M
4	2	11.5 ± 2.12	2 M

### AH collection and processing

The AH samples were obtained from 44 patients using a 26-gauge needle inserted into the anterior chamber of the eye according to previously reported methods [7]. Immediately after collection, the aqueous humor was transferred to dust-free Eppendorf tubes containing a protease inhibitor cocktail. The samples were then centrifuged at 1500 × g for 15 min at 4°C. The supernatant was collected and centrifuged again at 15000 × g for 15 min at 4°C to extract proteins and then stored at −80°C for further analysis.

### Proteomic profiling using iTRAQ labeling and LC-MS/MS

**Fig 1:**
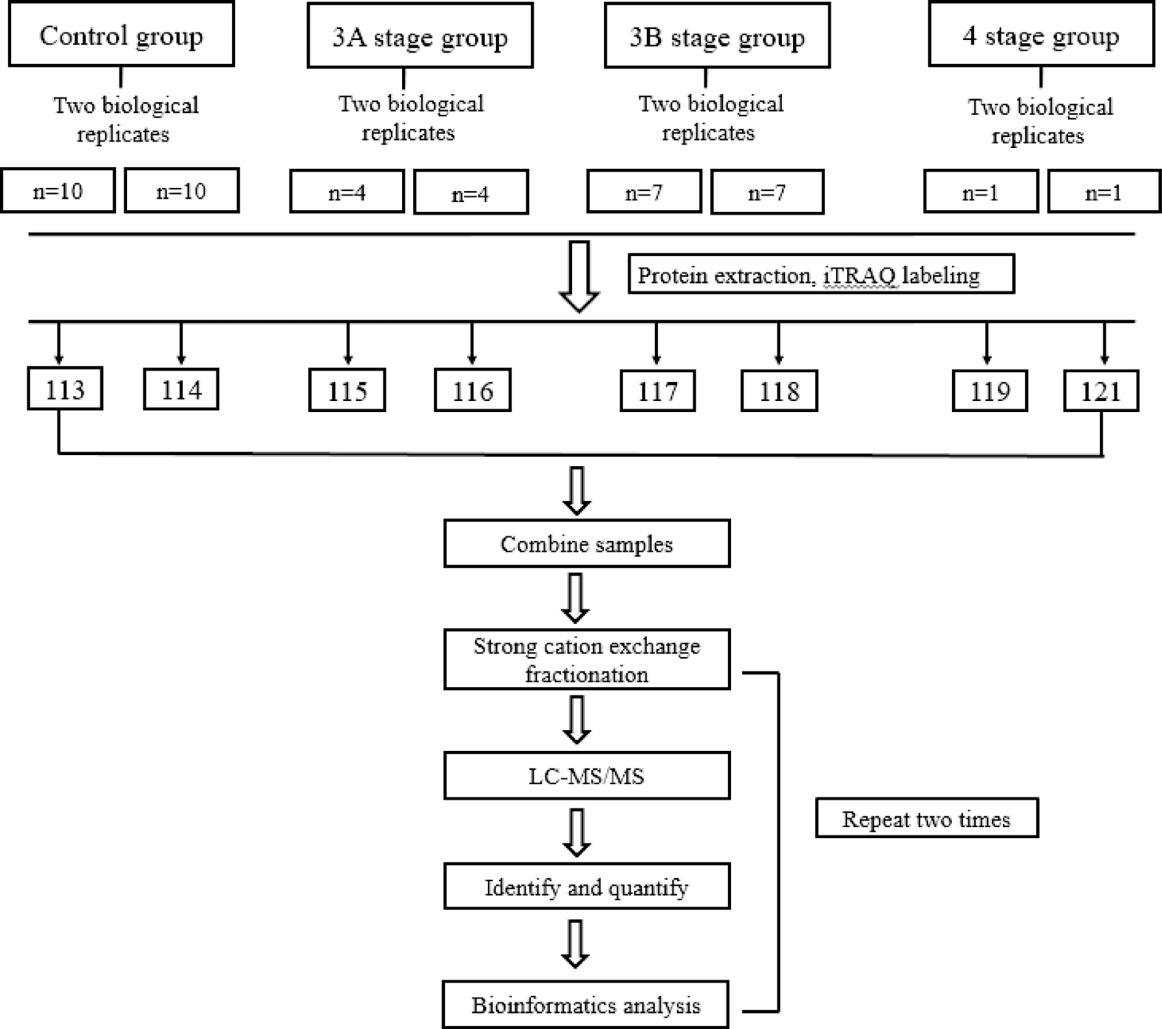
Schematic representation of the workflow of the iTRAQ experiment.

ITRAQ labeling was conducted according to previously reported methods [21]. Briefly, the protein samples from each group were harvested using cold acetone, digested with trypsin overnight at 37°C, and then subjected to iTRAQ labeling. The proteins extracted from the aqueous humor samples were divided into two duplicates and labeled as 113–114 (control), 115–116 (stage 3A), 117–118 (stage 3B) and 119–121 (stage 4) using an iTRAQ Reagent 8-Plex kit (Shanghai AB Sciex Analytical Instrument Trading Co., Shanghai, China) and then vacuum dried. A summary of the workflow performed in the current study is illustrated in Fig 1.

Subsequently, the labeled peptides were separated using strong cation exchange (SCX) chromatography and collected in ten SCX fractions. Each SCX fraction was analyzed on a Triple TOF 5600 system (AB Sciex) in duplicate and expressed as run 1 and run 2. The mass spectra of each iTRAQ labeled sample were obtained in an information-dependent acquisition mode. The mass range was set as 100–2000 m/z for the product ion spectra, and the reporting iTRAQ tags (113 and 114 m/z) were enhanced for quantitation. The ratio of the iTRAQ reporter ion intensities were used to measure the relative amount of the peptides and proteins in each sample according to previously published methods [22, 23]. Database searches were conducted using ProteinPilot Software version 4.5.

Proteins that presented a fold change greater than 2.0 and a p-value less than 0.05 between the patients and CK were considered differentially expressed proteins (DEPs). Hierarchical clustering of the selected probe sets was performed using a Pearson’s correlation analysis for the distance matrix and the Ward's linkage.

### Bioinformatics analysis of identified proteins

The DEPs were annotated using the GO database (http://www.geneontology.org/), and the protein classification was performed based on functional annotations using Gene Ontology (GO) terms for cellular components, biological processes and molecular functions. In addition, 46 identified differentially co-expressed proteins (DCPs) were assigned a gene symbol (Table 2) using the Panther database (http://www.pantherdb.org/). We analyzed the DCPs between the patients and CK and calculated a significance value for the GO categories using a cut-off criterion (*p* < 0.05).

**Table 2:** DEPs between the patients with stage 3A, 3B and 4 Coats’ disease and the controls (*p* < 0.05).

3A vs. CK	3B vs. CK	4 vs. CK	DCPs
93	125	146	46

### Western blotting

Western blot analyses were performed on AH samples to validate the results obtained by iTRAQ for some of the proteins that showed significant differences. In brief, equal amounts of sample (20 μg) were loaded on a 10% sodium dodecyl sulfate polyacrylamide gel. The proteins were then transferred into a polyvinylidene fluoride (PVDF) membrane (Bio-Rad, California, USA). After blocking with 2% bovine serum albumin, the membranes were probed with monoclonal antibodies of haptoglobin (sn: ab13429) and apolipoprotein C-I (sn: ab198288) (Abcam, Cambridge, MA, USA) followed by secondary antibody. The signal was then detected by enhanced chemiluminescence using LAS4000 system (GE, Fairfield, Connecticut, USA).

## Results

### Image acquisition and description of retinal morphology in Coat' disease

**Fig 2:**
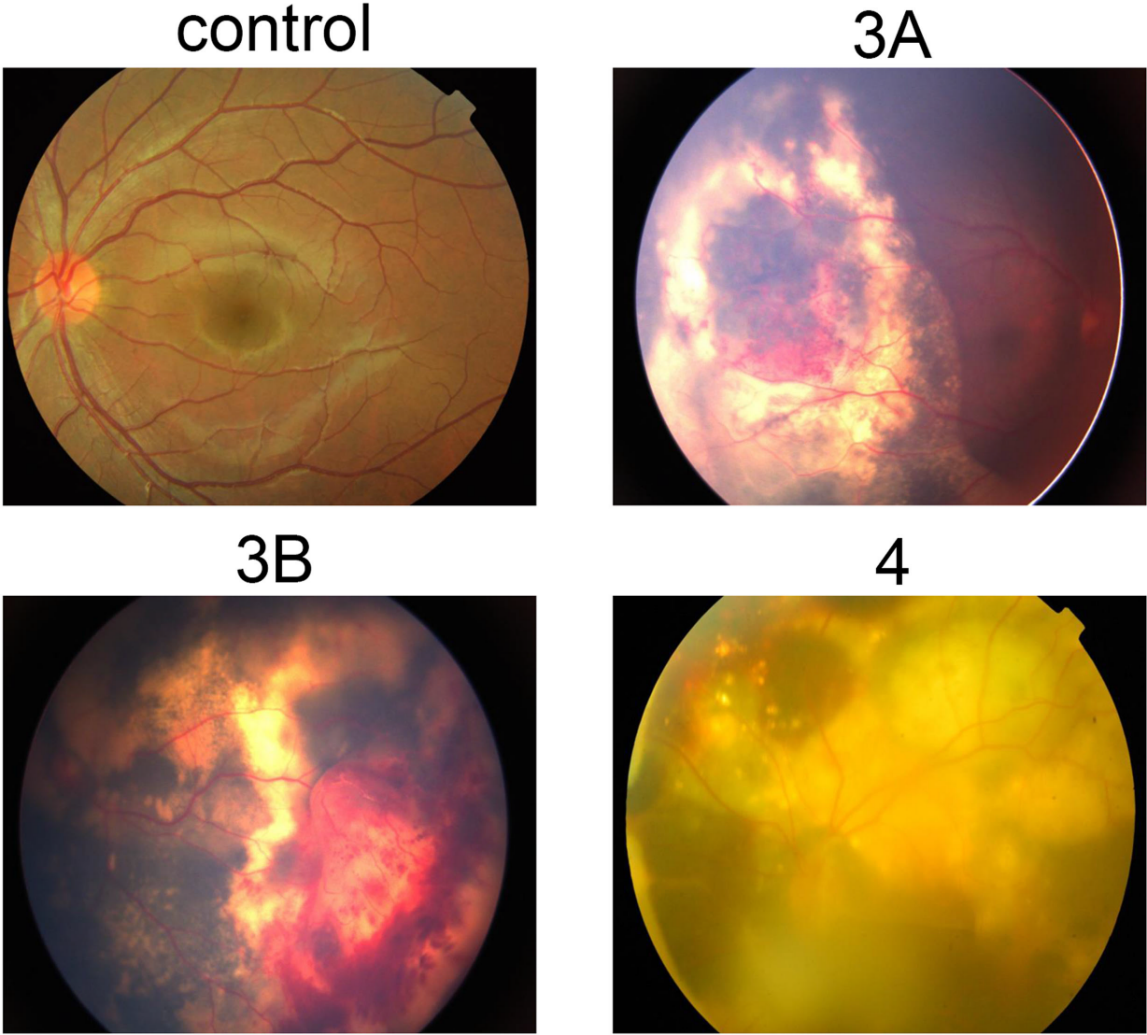
Representative fundus views of the different stages of Coats' disease, including stage 3A, stage 3B, and stage 4, and the controls.

After full dilation of the pupils, retinal photographs were obtained from patients with different stages of Coats' disease and the control participants using a Zeiss FF450+ fundus camera (Zeiss, Germany; and Imedos Systems, Germany). As shown in Fig 2, the control group did not present abnormal vascular changes. However, the fundus examination revealed that stage 3A patients presented with subtotal exudative serous retinal detachment and stage 3B patients presented with total yellow exudative retinal detachment. Compared with stage 3A, additional subretinal fluid and hyperpigmentation were observed in stage 3B. Retinal detachment accompanied by a large number of abnormal expansions of retinal blood vessels and retinal surface bleeding were observed in stage 4 patients (Fig 2).

### Proteomic analysis to detect differentially expressed proteins in the aqueous humor

**Fig 3:**
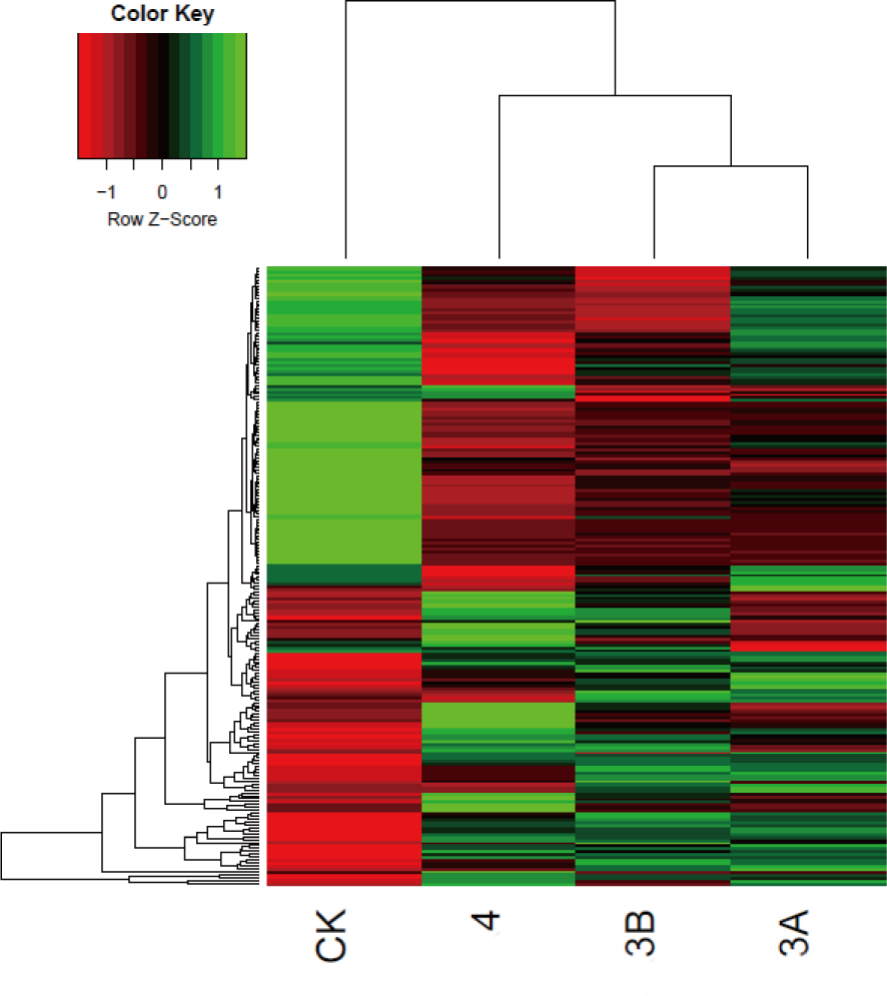
Heat map analysis of the differentially expressed proteins between the CK group and three groups of patients with different stages of Coats' disease. The color scale shown at the top illustrates the relative protein expression level across all the samples. Red represents an expression level lower than the mean, whereas green represents an expression level above the mean.

To investigate the mechanisms leading to the development of Coats' disease, a high-throughput quantitative proteomics analysis using iTRAQ labeling was performed to profile the aqueous humor from the patients presenting with three stages of Coats' disease and the control patients. The results of the iTRAQ analysis revealed that the protein profiles of the three stages of Coats' disease were significantly different. Of the total 819 identified proteins, 222 were significantly differentially expressed between the Coats' disease patients and the controls (S1 and S2 Tables). The hierarchical clustering heat map of the 222 DEPs is shown in Fig 3.

### Proteomic alterations in the aqueous humor of Coats' disease patients

**Fig 4:**
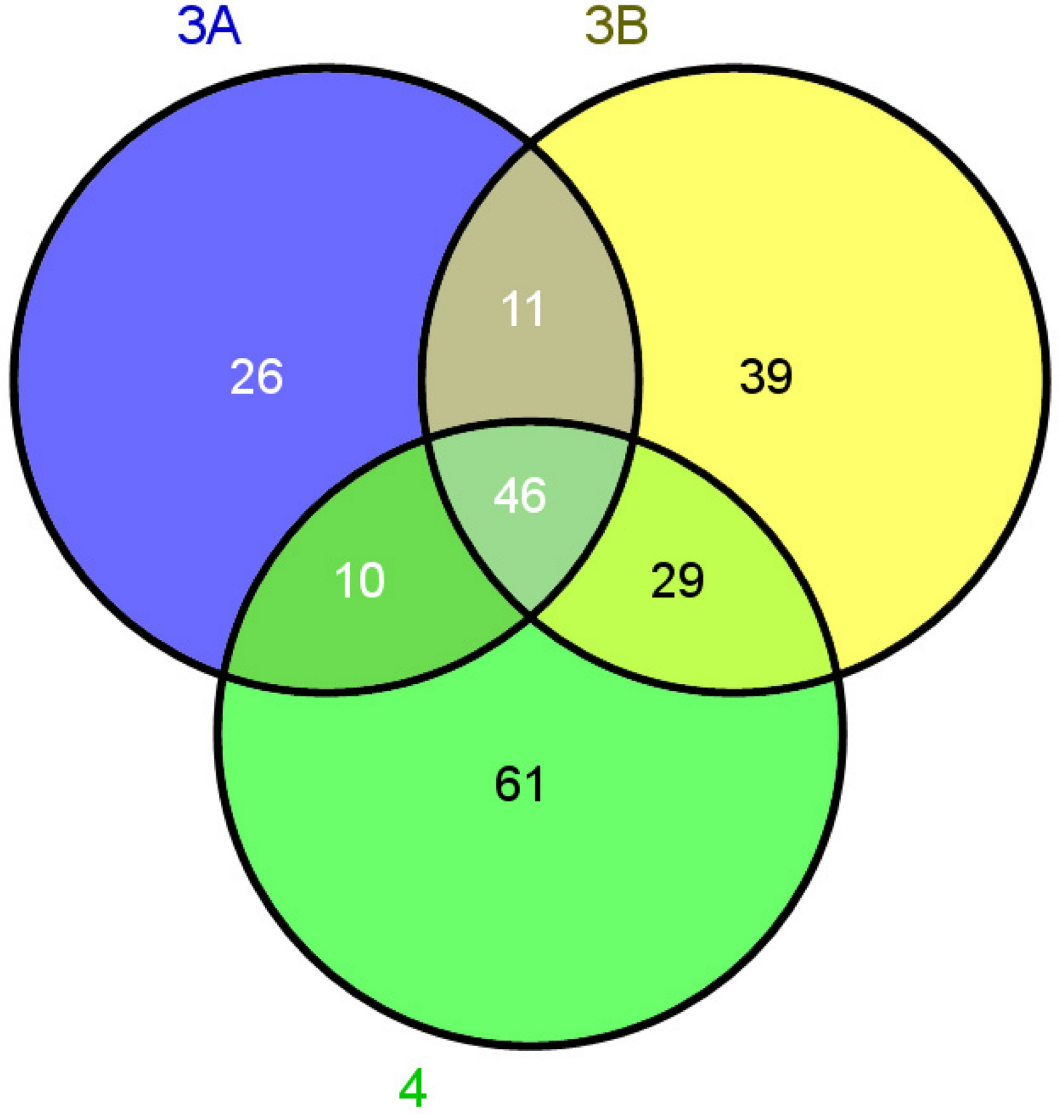
Venn diagram indicating the differentially co-expressed proteins (DCPs) from the patients with the three stages of Coats' disease. The numbers in parentheses indicate the total number of DCPs in stage 3A, stage 3B and stage 4, which are represented by purple, yellow and green, respectively.

We then identified the proteomic differences between the patients with stage 3A, 3B and 4 of Coats' disease and the CK group. As shown in Table 2, 93 proteins were differently expressed between the stage 3A patients and the CK group. Similarly, we found 125 and 146 DEPs between the stage 3B patients and stage 4 patients and the CK group, respectively. Moreover, a Venn diagram was used to analyze the DCPs between the three stages of Coats' disease and the CK group (Fig 4). The results showed that 46 proteins were common between each stage of Coats' disease and the controls. Among those, 17 proteins were up-regulated and 29 proteins were down-regulated in the patients with Coats' disease (Fig 4 and Table 3).

**Table 3:** Forty-six DCPs, including 29 down-regulated proteins (log FC < -1) and 17 up-regulated proteins (log FC > 1).

Gene symbol	Protein Name	Log FC*
*FGB*	Fibrinogen beta chain	4.08
*CFH*	Complement factor H	3.14
*FN1*	Isoform 10 of Fibronectin	3.23
*ITIH4*	Inter-alpha-trypsin inhibitor heavy chain H4	3.31
*FGG*	Isoform Gamma-A of Fibrinogen gamma chain	4.27
*HP*	Haptoglobin	4.28
*IGHA1*	Ig alpha-1 chain C region	3.17
*AFM*	Afamin	3.15
*IGHM*	Isoform 2 of Ig mu chain C region	3.65
*LUM*	Lumican	2.26
*SERPINA7*	Thyroxine-binding globulin	1.67
*VTN*	Vitronectin	2.77
*ITIH1*	Inter-alpha-trypsin inhibitor heavy chain H1	1.65
*APOC1*	Apolipoprotein C-I	3.96
*SAA4*	Serum amyloid A-4 protein	1.41
*F12*	Coagulation factor XII	1.54
*S100A6*	Protein S100-A6	1.51
*RBP3*	Retinol-binding protein 3	-4.06
*TTR*	Transthyretin	-1.58
*CTSD*	Cathepsin D	-3.14
*CST3*	Cystatin-C	-2.12
*MYOC*	Myocilin	-3.47
*GPX3*	Glutathione peroxidase 3	-2.64
*CRYBB1*	Beta-crystallin B1	-4.14
*SEMA7A*	Semaphorin-7A	-3.06
*CPE*	Carboxypeptidase E	-2.77
*C1S*	Complement C1s subcomponent	-1.64
*ENPP2*	Isoform 3 of Ectonucleotide pyrophosphatase/phosphodiesterase family member 2	-1.89
*CRYBB2*	Beta-crystallin B2	-3.91
*CLSTN1*	Isoform 2 of Calsyntenin-1	-2.06
*CRYGS*	Beta-crystallin S	-5.42
*SOD3*	Extracellular superoxide dismutase [Cu-Zn]	-2.94
*B3GNT1*	N-acetyllactosaminide beta-1,3-N-acetylglucosaminyltransferase	-1.77
*FAM3C*	Protein FAM3C	-3.18
*CDH2*	Cadherin-2	-2.47
*CRYGD*	Gamma-crystallin D	-3.64
*WIF1*	Wnt inhibitory factor 1	-2.37
*COL9A1*	Collagen alpha-1(IX) chain	-2.42
*IGFBP6*	Insulin-like growth factor-binding protein 6	-1.37
*CRYBA1*	Beta-crystallin A3	-4.23
*MFAP4*	Isoform 2 of Microfibril-associated glycoprotein 4	-2.00
*SEMA4B*	Semaphorin-4B	-2.14
*ASAH1*	Isoform 2 of Acid ceramidase	-2.61
*CTSA*	Lysosomal protective protein	-1.77
*ALDH1A1*	Retinal dehydrogenase 1	-1.89
*IMPG1*	Interphotoreceptor matrix proteoglycan 1	-3.32

* log FC, log_2_ of the average protein level fold change (FC) in the samples from stage 3A, stage 3B and stage 4 Coats’ disease patients.

### Functional analysis of the identified Coats' disease-related proteins

**Fig 5:**
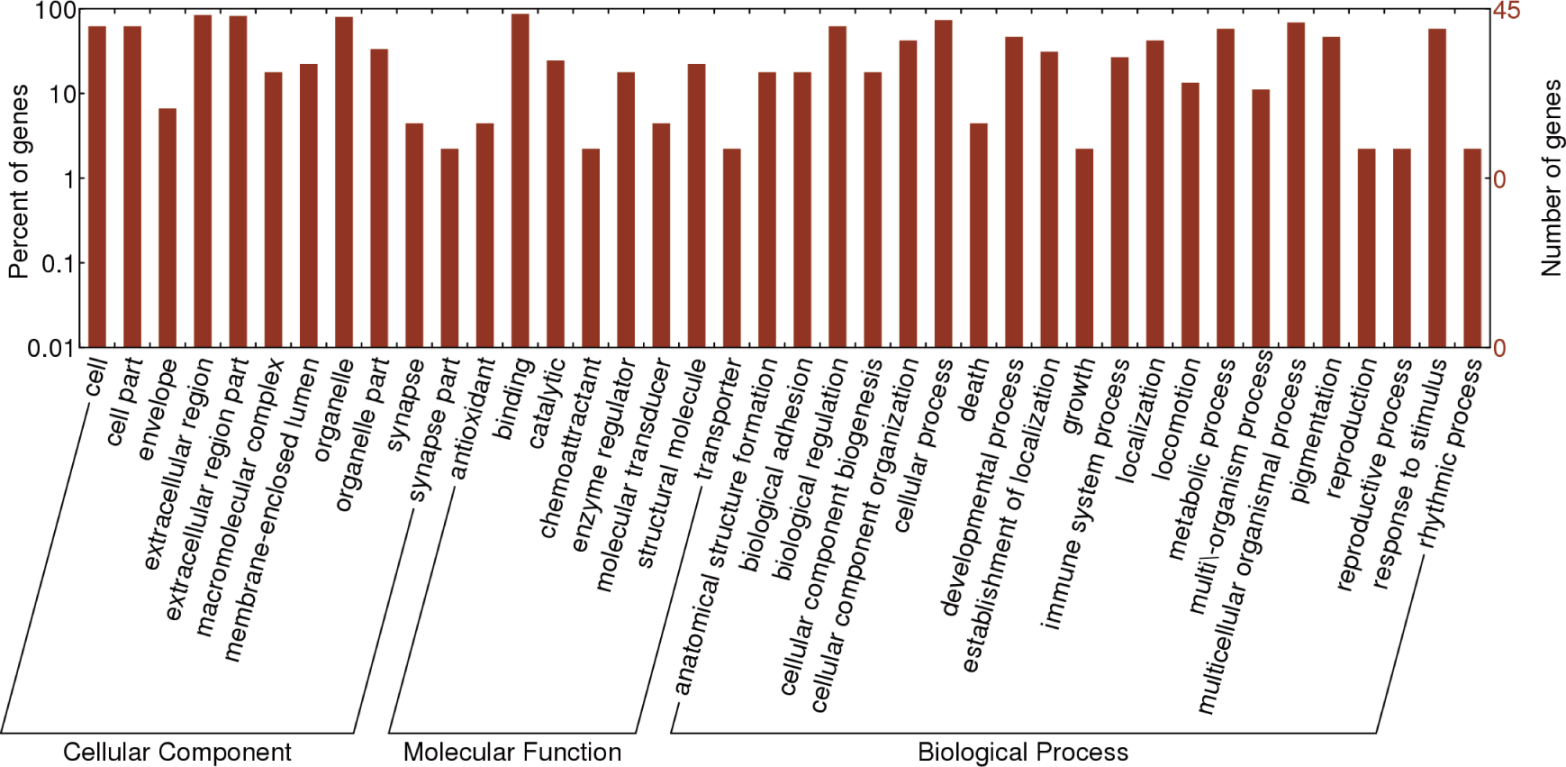
GO annotation and functional classification of the differentially expressed aqueous humor proteins using GO terms for cellular components, molecular functions and biological processes.

To investigate the functional significance of the DEPs identified in the patients with Coats' disease, 222 DEPs were functionally divided into three groups (cellular components, molecular functions and biological processes) according to the GO annotation analysis (Fig 5). Further analysis (Table 4) revealed that a total of 46 DCPs with a p-value of less than 0.05 were enriched for 6 GO terms. Crystalline lens related proteins, including βB1-crystallin (CRYBB1), βB1-crystallin (CRYBB2), γS-Crystallin (CRYGS) and γD-Crystallin (CRYGD), were found to play a role in structural molecule activity (*p* = 1.56E-06); myocilin (MYOC) was found to play an important role in protein binding (*p* = 0.00089) and receptor binding (*p* = 0.00081); apolipoprotein C-1 (APOC1) was found to be essential for enzyme regulator activity (*p* = 0.0009); and β-fibrinogen (FGB) was found to be closely associated with most GO terms.

**Table 4:** GO term classification of the DCPs between the patients and CK with *p*-values < 0.01.

Term	Biological Process description	Protein number	p-value	Genes
GO:0005196	structural molecule activity	10	1.56E-06	COL9A1/CRYBA1/CRYBB1/CRYBB2/CRYGD/CRYGS/FGB/FGG/IMPG1/LUM
GO:0050839	Cell adhesion molecule binding	6	2.67E-06	CPE/FGB/FGG/FN1/VTN/SEMA7A
GO:0005102	Receptor binding	10	0.0008104	FAM3C/FGB/FGG/FN1/IGHA1/IGHM/MYOC/TTR/VTN/SEMA7A
GO:0030234	Enzyme regulator activity	8	0.0009351	CST3/ALDH1A1/FN1/APOC1/ITIH1/ITIH4/CTSA/SERPINA7
GO:0070011	Peptidase activity, acting on L-amino acid peptides	6	0.0024658	CPE/CTSD/F12/CTSA/RBP3/C1S
GO:0008233	Peptidase activity	6	0.0028979	CPE/CTSD/F12/CTSA/RBP3/C1S

### Western blot

**Fig 6:**
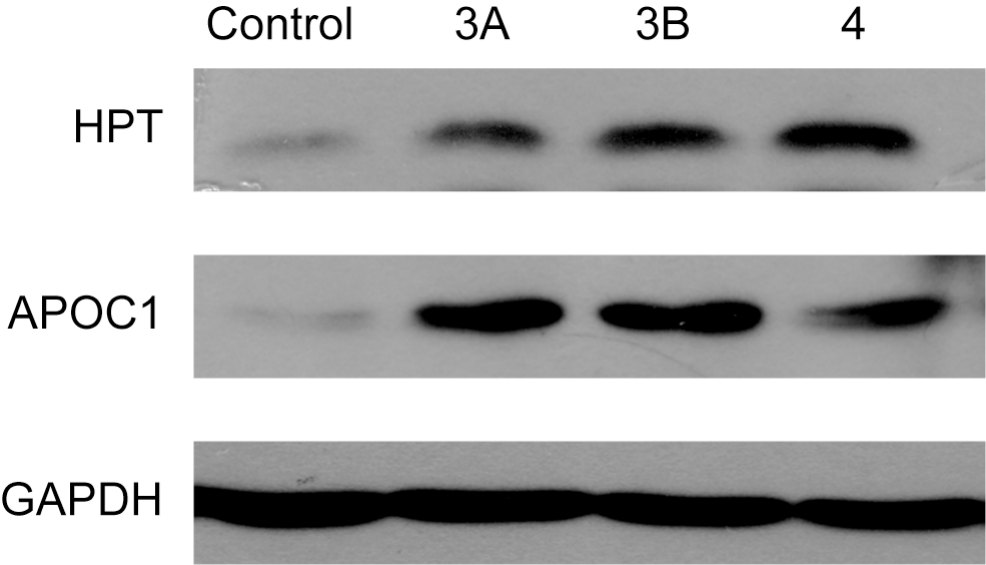
Western blotting analyses of 2 DCPs confirmed that the proteins accumulated in three groups of patients.

Because of difficulty in acquiring antibodies as well as the limited volume of AH samples, Western blotting was performed for only two of the DCPs, haptoglobin (HPT) and apolipoprotein C-I (APOC1). The results were shonw in Fig 6 and confirmed the up-regulation of these two proteins in Coats’ disease, indicating the potential value of DCPs we identified for further validation and investigation.

## Discussion

Coats' disease most commonly affects young boys who are often too young to report their symptoms [24]. In our study, 36 patients diagnosed with Coats' disease were classified based on three stages of the disease: stage 3A, stage 3B and stage 4. Most of the patients were between 10 and 15 years of age, which suggests that Coats' disease predominantly affects young males. To identify novel proteins associated with the pathogenesis of Coats' disease, we compared the AH proteins between patients with different stages of Coats' disease and control participants. A heat map analysis showed that the protein profiles from the patients with the three stages of Coats' disease were different from those of the controls, which indicated that the protein content in the AH changes during the development of Coats' disease. Furthermore, we screened 46 DCPs identified between the groups of patients with the three stages of Coats' disease and the controls and found 17 up-regulated proteins and 29 down-regulated proteins in the disease groups compared with the controls. The GO functional enrichment analysis indicated that most of the DCPs were closely correlated with structural molecule activity and molecular functions.

Of the identified DCPs, several crystalline-related proteins, including CRYBB1, CRYBB2, CRYGS and CRYGD, were down-regulated in the AH from patients with Coats' disease. Previous studies have shown that crystalline genes are closely related to lens opacity and microcornea [25]. CRYBB1 accounts for 9% of the total soluble crystalline in the human lens [26, 27] and is a critical protein in the formation of acidic β-crystallin heteromers in the lens. The formation of heteromers is considered important for the maintenance of lens transparency [28]. CRYGS is also important for maintaining lens transparency, and a dysregulation of CRYGS is associated with lamellar cataracts [29, 30]. Therefore, our results suggest that the down-regulation of crystalline-related proteins may play a crucial role in the occurrence of Coats' disease. To the best of our knowledge, vitamin A has diverse biological functions that include sensing light for vision, and the aldehyde form of vitamin A functions as the chromophore for visual pigments in the eye [31, 32]. Plasma RBP is a component of the outer segments of photoreceptors and can mediate cellular vitamin A uptake [33, 34] and prevent the potentially cytotoxic effects of retinoids. In addition, the presence of sufficient quantities of functional RBP3 is critical for photoreceptor survival [35]. The trabecular meshwork (TM) represents the anatomic location with the highest resistance to AH outflow [36]. MYOC, a secreted protein found in human TM, is considered a key regulatory node that governs extracellular matrix turnover homeostasis in the TM [37]. The down-regulation of MYOC promotes AH outflow, which elevates intraocular pressure [38] and may induce the occurrence of Coat's disease. We found that crystalline-related proteins, vitamin A-related proteins and TM skeleton proteins were down-regulated in the AH samples from patients with Coats' disease.

Interestingly, several genes, including APOC1 and FGB, were up-regulated in the AH samples of the patients with Coats' disease (Fig 6). Further analyses indicated that APOC1 is a component of multiple lipoproteins and regulates lipid metabolism and transport [39]. The overexpression of human APOC1 has been demonstrated to produce hyperlipidemia and increase serum lipid levels [40, 41]. Furthermore, APOC1 is closely associated with vascular inflammation and retinitis pigmentosa [42], and reports have indicated that FGB is closely related to elevated plasma fibrinogen levels and ischemic stroke [43, 44].

In summary, our findings revealed that the proteomic composition of AH significantly differed between individuals with Coats' disease and the controls and the proteins identified in this study could serve as potential biomarkers for Coats' disease. In addition, we suggest that the DCPs identified in the patients with Coats' disease contribute to the pathologic changes and complications of Coats' disease. However, further investigations are necessary to explore the exact mechanism by which these DCPs underlie the occurrence of Coats' disease.

## Supporting Information

S1 Table Detailed information of the 819 identified proteins (ProteinPilot score > 1.3). (XLSX)

S2 Table Quantitative and descriptive data for the 222 significantly differentially expressed proteins between the Coats' disease samples and control samples. (XLSX)
